# Navigating No Recourse to Public Funds and NHS maternity charging: health and social care professionals’ experiences of supporting pregnant women in the UK

**DOI:** 10.3389/fpubh.2026.1772211

**Published:** 2026-05-22

**Authors:** Tisha Dasgupta, Sahla Ansari, Lydia Mengistu, Rebecca Sobodu, Chiamaka Elumogo, Siofra Peeren, Sam Burton, Zenab Barry, Kirsty Kitchen, Hannah Rayment-Jones

**Affiliations:** 1 Department of Women & Children’s Health, School of Life Course and Population Sciences, King’s College London, London, United Kingdom; 2 School of Neuroscience, Institute of Psychiatry, Psychology & Neuroscience, King’s College London, London, United Kingdom; 3 Department of Global Health & Social Medicine, School of Global Affairs, King’s College London, London, United Kingdom; 4 Division of Methodologies, Florence Nightingale Faculty of Nursing, Midwifery & Palliative Care, King’s College London, London, United Kingdom; 5 Lambeth Health Determinants Research Collaboration, Lambeth Council, London, United Kingdom; 6 School of Psychology, Liverpool John Moores University, Liverpool, United Kingdom; 7 Birth Companions, London, United Kingdom

**Keywords:** health and social care, inequality, maternity care, No Recourse to Public Funds, professionals

## Abstract

In the UK, many migrant women are subject to the No Recourse to Public Funds condition, which restricts access to welfare and can result in liability for maternity care charges, contributing to poverty, delayed care, and health inequities. This mixed-methods study examined health and social care professionals’ knowledge, confidence, resources, and training needs when supporting women with this status during pregnancy and early motherhood. Focus groups with 16 health and social care professionals and an online survey of 65 professionals and students were conducted across maternity, social care, and voluntary sector settings in England; data were analysed thematically and descriptively. Findings showed wide variation in the support provided, driven by limited understanding of immigration rules, available perinatal support, and maternity charging, alongside inconsistent access to specialist advice. Professionals reported low to moderate confidence in supporting women and responding to charging-related questions, with substantial variation in preparedness across roles and settings. Although most respondents wanted to improve their understanding, only 14% had received prior training. These findings highlight how policy complexity and workforce knowledge gaps may contribute to inconsistent care and inequities during pregnancy and early motherhood, underscoring the need for clearer guidance, workforce training, and improved cross-sector coordination. The resulting realist programme theory will inform co-designed, evidence-based guidance and training relevant to public health systems supporting migrant populations.

## 1 Introduction

In the United Kingdom (UK), individuals who fall within certain immigration-related parameters can be described as having “No Recourse to Public Funds” (NRPF) (1). These “public funds” encompass a range of benefits (including Universal Credit, and Child Benefits), social housing provisions, and specific types of NHS treatment (2). As of 2025, an estimated 3.3 million people were subject to NRPF (3), disproportionately affecting those from Black, Asian, and other minority ethnic backgrounds. There is a roughly equal split of men and women with NRPF immigration status, however, there is limited data on the number of pregnant and postpartum women and birthing people with NRPF. Families with NRPF are also at high risk of deep poverty and hardship: studies have found that a majority of such households struggle to afford essentials like food, heating and bills, with rates of going without basics and falling behind on bills much higher than for other low-income families, and many families with children reporting hunger and material hardship (4). Research from the UK (5–7) and globally (8) show migrant women and children are subject to exclusionary healthcare and immigration policies that introduce additional barriers. They are more likely to attend maternity services later than recommended and receive inadequate care, while being less likely to attend maternity triage or give birth in hospital settings (5). They are at a disproportionate risk of adverse perinatal outcomes for both mother and baby, indicating the “healthy migrant effect” may not apply during the perinatal period (6–8).

Although under current UK Government regulation maternity healthcare is classified as essential and free at the point of use (9), its designation as secondary care means that many with NRPF are still subject to NHS charges. This includes fees at 150% of standard NHS tariffs, and unpaid debts can be reported to the Home Office, warranting investigating into individuals’ immigration statuses. Although NHS Trusts have a duty to employ a named Overseas Visitor Manager, tasked with implementation of charging policies, unless exempt such has having paid a health surcharge upfront prior to obtaining a visa, guidance is often vague or inconsistently applied (10).

A lack of standardised guidelines and policies can lead to anxiety and ambiguity when put into practice, for service users and providers alike. A 2020 report uncovered that several Trusts were found to have no policy for the practical application of charging practices at all (11). Previous qualitative research into the implementation of NRPF policy in general healthcare settings has highlighted themes of institutional mistrust, disconnect between policy and practice, and communication difficulties (12). However, there is a significant gap in knowledge regarding how these dynamics operate in maternity care specifically—particularly in how NRPF policy is understood and enacted by professionals who work with pregnant migrants, and the extent to which these policies impact access, safety, and equity of care (13).

Furthermore, much of the existing research does not account for the nuanced and heterogeneous nature of the population affected by NRPF and NHS charging. Not all migrant women are equally impacted—charging practices differ based on visa type, documentation status, and service location (14). There is also a lack of data on how misinformation, variation in staff knowledge, and fragmented guidance influence how care is provided and women’s experiences and outcomes.

This study addresses these gaps by exploring professionals’ views on the practicalities of supporting pregnant women with NRPF who may be subject to NHS charging policies in England, and by considering perspectives surrounding existing policies and programmes, knowledge gaps, training needs, and ideas to facilitate improved care. We aimed to explore the experiences of professionals working with pregnant women and families who have NRPF status in the UK, in order to assess availability of resources, current knowledge base, and future training needs to improve care experiences and outcomes.

## 2 Methods

An exploratory sequential study design was adopted using a pragmatic approach to explore professionals’ lived experiences of supporting women and families with NRPF, preliminary findings of which then deductively informed a quantitative survey. This approach was taken as there is limited research on professionals’ knowledge and experience of caring for NRPF service users, and variability in existing guidance across England. As such as it was crucial to understand the key areas of interest first.

### 2.1 Sampling, recruitment, and participants

Purposive sampling was used to recruit professionals currently working with pregnant women and families with NRPF status in England, for online focus group discussions. Participants were purposively selected from a range of sectors, including healthcare, social work, local authorities, charities, legal services, and community organisations. Efforts were made to ensure geographic diversity, targeting regions with high migrant populations. Recruitment was conducted through online advertisements shared via the research team’s existing networks and collaborations (e.g., working groups, professional contacts), as well as through relevant charities such as Maternity Action, Birth Companions, Project 17, and The Happy Baby Community. Eligibility was assessed (by TD) using a screening questionnaire completed prior to the focus groups, which gathered basic demographic data and confirmed written consent. Inclusion criteria were: over 18 years of age, English-speaking, working in England, and currently supporting women and families with NRPF.

A total of 16 participants were recruited across six focus group sessions, conducted between November 2024 and March 2025. Study sample size was reflected and decided upon based on information power, concepts aligning with the principles of the qualitative study design, when no new themes were emergent and meaning saturation was achieved. Demographic information is presented in Table 1.

**Table 1:** Focus group participant demographics.

Participant	Profession/ role	Organisation	Years in role	Geographic region
P01	Social worker	LA	<2	London
P02	HCP, Midwife	NHS	6–10	London
P03	HCP, Midwife	NHS	11+	London
P04	Social worker	LA	2–5	Yorkshire and the Humber
P05	Manager	LA	2–5	London
P06	HCP, Midwife	NHS	11+	London
P07	Social worker	LA	<2	London
P08	HCP, Midwife	NHS	<2	South East
P09	HCP, Midwife	NHS	6–10	North East
P10	Co-founder	Charity	2–5	West Midlands
P11	Manager	LA	6–10	London
P12	HCP, Midwife	NHS	<2	South West
P13	Support worker	Charity	2–5	London
P14	Support worker	Private care service	2–5	West Midlands
P15	Manager	Charity	11+	North West
P16	Solicitor	Charity	6–10	UK wide

LA, local authority, HCP, healthcare professional, NHS, National Health Service.

Using the same recruitment strategy, a further 78 participants completed an anonymous online survey to better understand professionals’ understanding and confidence levels surrounding NRPF. Participants were directed to the survey if they were above 18 years of age, worked in any capacity with pregnant women or families with NRPF in England. Recruitment was conducted using professional networks, charities, staff newsletters, and social media. A prize draw was facilitated to encourage participation.

Responses with more than 80% of data missing were removed *a priori*, leaving 65 responses for analysis. Table 2 presents demographic details of survey participants.

**Table 2:** Online survey participant demographics.

Baseline characteristic	*N*; total *n* = 65	%
Ethnicity		
White/ White British	49	75.4
Black/Black British	6	9.2
Asian/ Asian British	5	7.7
Mixed/ Multiple ethnic groups	2	3.1
Other ethnic group	3	4.6
Professional organisation		
NHS	56	86.2
Social work	4	6.2
Other	5	7.7
Years in profession		
<2 years	6	9.2
2–5 years	8	12.3
6–10 years	10	15.4
11 + years	41	63.1
Workplace geographic region		
London	38	58.5
South West	8	12.3
Yorkshire and the Humber	6	9.2
North West	4	6.2
North East	2	3.1
West Midlands	2	3.1
East Midlands	1	1.5

NHS, National Health Service.

### 2.2 Data collection

Focus groups were considered an appropriate method of data collection to not only collect individual experiences and the opinions of each individual but also to provide insights into mechanisms of working with other professionals as is common in supporting women with NRPF. Six focus groups were carried out online via Microsoft Teams video conference. These were conducted by an experienced qualitative researcher [TD] and facilitated by one other study team member [HRJ, SP, RS, CE] who took field notes. The semi-structured interview guide (see Supplementary file 1) with guiding questions was developed by the multidisciplinary team and reviewed by the PPIE lead of the NoRePF study [ZB]. Focus groups were recorded and transcribed by a third-party transcription service. Participants were asked about their general experience supporting pregnant women and families with NRPF, their knowledge of existing programmes and policies, and ideas for how future guidance or training can be delivered to enhance support for this community.

The anonymous survey was administered between April and November 2025 via the online survey platform Qualtrics XM™. The survey was divided into four main parts: (1) *Participant demographics*; (2) *Immigration and NRPF* which included questions on knowledge of immigration rules, NRPF as it pertains to maternity care, and of available eligible services, as well as confidence levels of supporting pregnant women with NRPF; (3) *NHS charging* which includes questions on understanding of NHS charging practices and confidence in asking and handling questions about being charged for maternity care; and (4) *Training* which included questions on if participants had ever received training related to NRPF, whether they would take such training, and in what format it would be best received. Participants were asked to score each on a 0–100 visual analogue scale (VAS), to allow greater variability in measures given the subjective nature of self-reporting knowledge and confidence, as well as to add any additional comments in free text responses at the end of each section.

### 2.3 Data analysis

Data from the focus groups were analysed using reflexive thematic analysis (15), which involves inductive coding practices which are consultative and open. NVivo 14 was utilised for data management and analysis which followed a six-stage approach. Familiarisation with the data involved repeated reading of the transcripts and initial note-taking to capture early impressions and ideas. Codes were generated inductively using NVivo software, with the aim of capturing meaningful features of the data relevant to the research questions. Themes were developed and named and renamed, through an iterative process of reviewing coded data, identifying patterns of shared meaning, and engaging in reflexive discussions amongst the research team. Each focus group transcript was coded by one researcher [TD, RS, CE] and reviewed collaboratively together with the study lead [HRJ]. Regular discussions were held between all researchers to reflect on arising concepts, resolve discrepancies, and revise analysis, coding, or themes, thereby enhancing analytical rigour. The analysis aimed to produce a rich thematic account of the entire dataset, using a largely inductive, data-driven coding process. Coding focused on the explicit, semantic content of the data, rather than latent or interpretative meanings. Reflexive notes were taken in each session and during analysis, to reflect on how the research team’s interdisciplinary background and lived experiences may have influenced or informed the research and its outputs. The role of multiple researchers involved in data collection, analysis, and interpretation was south as a means of enhancing interpretative depth and rigor, rather than for accuracy or reliability (16). Themes were generated based on their relevance and salience to the research question, rather than solely on how frequently they appeared in the dataset.

Quantitative survey data were exported from Qualtrics to Microsoft Excel for cleaning and data visualisation. R statistical software was used to analyse the survey Visual Analogue Scale (VAS) responses, using the tidyverse package (17). Descriptive analysis was conducted for each response. Free text responses were analysed thematically as described above (by LM, along with regular discussions with the wider team to refine coding) and mapped on to the thematic findings of the focus group data. We present the qualitative findings from the focus group and survey analysis integrated together below. The contributing data were analysed for complementarity and expansion of themes.

## 3 Results

### 3.1 Quantitative results: online survey

There were 65 total responses included for analysis (*n* = 13 removed for having >80% data missing), collected between April and November 2025. All but one question was answered by all 65 participants. On a self-rated 0–100 VAS, we asked women to rate their knowledge of immigration rules and regulations generally and specifically for maternity services, confidence supporting women and families with irregular immigration status during pregnancy and the postnatal period, and of NHS charging practices in maternity care. Overall, low to moderate knowledge and confidence was reported. Wide standard deviations indicated variable preparedness between participants.

Mean self-reported knowledge scores of general immigration rules and regulations were 52.2 (SD = 27.3), of NRPF visa condition specifically were 50.8 (SD = 30.0), and rights and support available to women during pregnancy and the postnatal period 45.3 (SD = 28.1). Mean confidence scores in asking women about their immigration status were 71.1 (SD = 30.7) but lower for supporting pregnant and postpartum women with NRPF status (57.1, SD = 36.2). Similarly, in regard to NHS charging practices, mean self-reported knowledge and confidence in asking women about their overseas status and eligibility for NHS maternity care was 43.4 (SD = 28.7) and 57.14 (SD = 33.8) respectively, but confidence in answering questions and providing support was 42.5 (SD = 32.5). Table 3 presents these details.

**Table 3:** Survey results related to knowledge and confidence.

Survey questions	*n*	Mean 0–100 (SD)
Rating knowledge of immigration rules		
Knowledge of immigration status, rules, and regulations	65	52.2 (27.3)
Knowledge of the NRPF visa condition	65	50.8 (30.0)
Knowledge of rights and support available during pregnancy and postnatal period	65	45.3 (28.1)
Rating confidence in supporting women with irregular immigration status		
Confidence in asking women about immigration status	64	71.1 (30.7)
Confidence in supporting women with NRPF or undocumented status during pregnancy and postnatal period	64	57.1 (36.2)
Rating knowledge and confidence related to NHS charging		
Knowledge of NHS charging practices for maternity care	65	43.4 (28.7)
Confidence in asking women about their overseas status and eligibility for NHS maternity care	65	57.1 (33.8)
Confidence in answering women’s questions about their overseas status or eligibility for NHS maternity care	65	42.5 (32.5)

SD, standard deviation.

Participants were also asked about training related to NRPF and NHS charging, as well as preferred modes of training (see Table 4). Although 89.2% (*n* = 58) wanted to improve their understanding of NRPF and NHS charging, only 13.8% (*n* = 9) had received prior training. The most commonly preferred modality to receive this training was through online resources (*n* = 44, 67.7%).

**Table 4:** Survey results related to training and development.

Survey questions	*n* (%); total *n* = 65
Ever received training related to immigration and NHS charging in maternity care	
Yes	9 (13.8)
No	52 (80)
Not sure	4 (6.2)
Need to improve knowledge and confidence	
Yes	58 (89.2)
No	3 (4.6)
Not sure	4 (6.2)
Preferred ways to improve knowledge and confidence	
Online resources	44 (67.7)
Training sessions	42 (64.6)
Workshops	34 (52.3)
Written guidelines	38 (58.5)
Specialist role/oversight	23 (35.4)
Personalised AI tool	12 (18.5)

### 3.2 Qualitative results: focus groups with professionals and free-text survey responses

Three main themes were identified to describe professionals’ experiences of working with pregnant women and families with NRPF status and/or those subject to NHS charging, comprising eight sub-themes to capture specific experiences (Table 5). This captures qualitative data from open-ended survey questions, as well as focus group discussions with *n* = 16 professionals. The participant sample showed limited diversity in key demographics: most survey respondents were White (75%) and employed within the NHS (86%), with a majority having over 11 years of experience. Focus group participants were concentrated in London and a few other regions, and ethnic and professional diversity was limited. This prevented us from drawing insights into differences in knowledge between groups. Each thematic section also presents solutions for improvement, as proposed in the focus group discussions. Exemplary quotations, integrating qualitative data from both focus group discussions and free-text survey responses, are presented in text to support thematic findings. Focus group participants are represented by a prefix ‘P’ and survey responses with ‘S’.

**Table 5:** Overview of main themes and sub-themes.

Main theme	Sub-themes
1.0 Ineffective cross-sector communication	1.1 Breakdown of communication between teams
	1.2 Lack of clear referrals and responsibility
	1.3 Difficulty asking about NRPF status
2.0 Lack of knowledge and resources	2.1 Insufficient or burdened services
	2.2 (Over)reliance on charities and individuals
	2.3 Variation across services
3.0 Systemic and legislative barriers	3.1 Dehumanised individuals
	3.2 Need for changes to legislation

#### 3.2.1 Theme 1: ineffective cross-sector communication

The first theme describes professionals’ challenges with communication, working across different sectors such as maternity care, local authorities, social care, and charities, when supporting pregnant women with NRPF status. To overcome these challenges, a streamlined communication channel by way of a named key-worker, was recommended by participants.

##### 3.2.1.1 Breakdown of communication between teams

Participants described how, due to the complex nature of NRPF status and the support individuals are eligible for, care was often handled by different members of different teams in multiple organisations. At the same time, they noted a lack of clear communication and cohesive working between these individuals.

Participants reported siloed working, often resulting in duplication of effort and excessive paperwork.

*“I don’t think they [different sectors] connect up, and I don’t think they communicate with each other, they’re very much in a silo, their own silo. And I think sometimes they can double up on services, without even realising that they’re doing it.”—P12, HCP*

*“So I think communication is really lacking… you spend so much time chasing and on paperwork and kind of repeating things because you’ve referred somebody somewhere and then they don’t really do that anymore or they don’t exist, so you’ve got to do another referral.”—P06, HCP*

Some reported not knowing who to contact for help as there are lots of different people involved.

*“It’s lots of different people that are involved with the care and it is very fragmented … it’s hard to know who’s the person that’s pulling it all together.”—P10, Charity*

##### 3.2.1.2 Lack of clear referrals and responsibility

Participants highlighted that a lack of clear pathway for support and communication meant that women were often referred to a service incorrectly, which did not have capacity or resources, or were not equipped to handle such a case.

*“But there was a referral into social services, there was nothing they said they could do to house her, and [said] ‘if she came from wherever, could she not return?’”—P03, HCP*

*“Whilst I can see why local authorities don’t want to pick this up, it’s primarily because there is no exit plan of finances. So we don’t know clearly from the Home Office as to how long their decision making is going to take for these families to start regularising their immigration. So local authorities are a little bit wary to touch this, because actually where does it end?”—P05, LA*

*“I assume someone like the community midwife will be asking these questions.”—S16, HCP*

Participants reported a sense of passing off responsibility and blaming other services.

*“With the councils, I've had some really difficult experiences where it's just felt like they’ve just wanted …. [pause] they've just wanted the women with NRPF to be like ‘not their problem anymore’ and just kind of want to wash their hands of them.”—P8, HCP*

##### 3.2.1.3 Difficulty asking about NRPF status

The process of determining service users’ NRPF status and the support available to them was also described as being impacted by the complexity of the system, often resulting in difficult care interactions.

*“And I think our knowledge is not always great, as we know the migration system, it’s really, really complicated. And I think people, a lot of midwives just don't understand and don't know what to ask. I feel like I can quite quickly get to the bottom of someone's status, and sometimes it's not about asking. You know, you're not going to say, “Do you have No Recourse to Public Funds?” You have to kind of ask questions a bit of a different way, more sensitively and also in a different way.”—P8, HCP*

Participants reported having to be honest with women about the consequences of discussing NRPF status, including that, even when women were not directly referred to overseas charging offices, they might still be charged for their maternity care or referred to other services such as social care.

*“She did ask about being charged for her maternity care, and I was really direct and honest with her. I said “Being honest with you, I won't personally refer anyone to our overseas department. That's just my decision. I don't feel it's my place. However, I can't guarantee that you won't somehow get referred to the overseas, so that may happen.”—P8, HCP*

They noted concern that some women may avoid accessing services or asking for help due to fears that doing so could negatively affect their visa application or their current immigration status.

*“But I think there’s quite a lot of fear as well about families reaching out for official support, because it’s almost like they’re worried that that might impact their visa, or if they claim something which actually then they’re not eligible for, they’re worried that’s going to end in deportation. So there is, I think, a bit of fear and resistance maybe.”—P12, HCP*

*“The thing is that women, when they give them [NHS charging] bills like that, they get scared and they don’t respond to the NHS. So I’ve learnt from the training I’ve had that just for them to acknowledge the bill, it will go a long way and it will avoid them being reported to the Home Office, and it will make them able to access more healthcare. Because when they keep quiet, it makes things worse for them.”—P13, Charity*

*“I don’t think I should as it means the woman will not trust me enough to tell me the truth about her individual needs.”—S8, HCP*

##### 3.2.1.4 Suggestions for improvement

To overcome these challenges, participants recommended establishing streamlined communication channels between stakeholders across sectors. One suggestion was the introduction of a named key worker to act as a liaison between teams.

*“I think there should be a lead, and I think it’s important for the lead—whoever the agency is it doesn’t really matter - but somebody should be kind of assigned as the lead person so that that person can then follow up and maybe do a face-to-face meeting, even just like once every 6 weeks during the pregnancy just to keep updates so everyone knows what’s going on.”—P6, HCP*

For the role to be effective, participants emphasised the individual had to be in a senior position and one who had a good understanding of different services.

*“It's got to be someone with enough like, seniority or, yeah, at a high enough level that it means that people will listen.”—P11, LA*

*“Also it would need to be a role where people, we've really looked at the experience that they had, so the ability to have an understanding of what women with NRPF may have gone through, but also of how the different systems work. Because we all work really differently and … Yeah, it could work. It would also be quite nice to have a point of contact, because I think, what can be a bit overwhelming for us, is working out where to go for what support. We sometimes get confused with where Children's Services begin and end when it comes to NRPF.”—P8, HCP*

#### 3.2.2 Theme 2: lack of knowledge and resources

The second theme is related to lack of knowledge of available services for pregnant women with NRPF status, which vary across the country, and lack of resources overall leading to services being stretched and burdened and relying on informal support from charities who often struggle with resources themselves. Participants acknowledged that charities play a vital role in the migrant health landscape but are in urgent need of increased funding.

##### 3.2.2.1 Insufficient or burdened services

Participants reported not knowing about existing services and resources, which were already described as extremely limited and disjointed, thereby affecting professionals’ ability to make referrals as required.

*“I know that there is a real lack of knowledge, partly just because people with NRPF have so few rights and entitlements that it can be really hard to navigate”.—P11, LA*

*“But I do feel a little bit where my hands are tied sometimes, and there’s very limited resource that I can signpost people to.”—P12, HCP*

*“Services and support services are often changing so it’s hard to keep up.”—S11, HCP*

Adding to this barrier, participants noted that existing services, particularly related to mental health support, which was deemed as crucial, were also extremely burdened with not enough staff to meet needs due to budget constraints and staff shortages.

*“And I mean, we have a trauma counsellor, she's completely overwhelmed with referrals. And we just don't have enough psychological support, particularly for those clients, when I think they need a lot of that.”—P6, HCP*

*“Yeah, mental health support is so, so crucial. And exactly the same, they're completely oversubscribed. The perinatal mental health team in [city] are referring to us all the time and I'm going, “I'm referring back to you.”—P10, Charity*

##### 3.2.2.2 (Over) reliance on charities and individuals

Although participants agreed that charities played a key role in the multi-agency support networks, due to the lack of sufficient formal resources and support, many struggled to find services with the capacity to help women, or had to rely on these already stretched services, and on donations from the public, to plug gaps in support.

*“So a lot of the time we rely on things like donations, and generosity of the local community, and things like that.”—P15, Charity*

*Most charities, their hands are even full now, when you want to refer to them, they’ll tell you they are run out of capacity, they can’t help. It’s difficult to communicate with them, you just have to be, let me say, resilient, continue calling them, letting them know the importance it has on these children or the family … go and look for another charity, because most charities, their hands are tied because the government is not helping them, and they’ve really run out of capacity to take new people.—P13, Charity*

Additionally, given the dire lack of resources that staff could direct women to, they often went above and beyond their professional capacity. They described how, over the long term, this could lead to increased emotional burden and burnout. Staff had to gain knowledge and experience externally.

*“I did most of her laundry, and bought her new clothes, so that when she attended appointments she can actually be presentable and not smell of the streets.”—P3, HCP*

*“You’re doing this work vocationally, so you want to do the job well. It’s not just, say, something that you can close the door at the end of the day and forget about.”—P10, Charity*

*“I volunteer for an asylum refuge charity, I’ve worked with a continuity team looking after migrant pregnant women.”—S43, HCP*

##### 3.2.2.3 Variation across services

A difference in knowledge and in the availability of services was observed between participants working in different regions. Participants attributed this to the difference in number of cases encountered annually; for example, a large Metropolitan city has a much higher proportion of women with NRPF compared to rural settings. Allocation of resources was also described as varied across the country, or “postcode lottery.”

*“I think the picture in the North is very different, these cases come around quite rarely, and when they do I would describe the general feeling amongst senior managers as panic [laughs], they don’t really know what to do or how to proceed.”—P4, LA*

*“I completely agree, because in [city] we have lots of different districts, so over in [local district], which is geographically five minutes, or not even that, you cross over a road and then you’re in a different district, and there is a completely different amount of resource, lack of actually, and it’s really, really noticeable. And it is a postcode lottery, completely.”—P12, HCP*

*“This is not something I have come across in my work often.”—S48, LA*

##### 3.2.2.4 Suggestions for improvement

Firstly, all participants agreed there needs to be more financial resources provided to existing services—statutory and voluntary sector - so that they can continue providing vital support to women and families with NRPF.

*“I would love to see that NGOs get some money injected into them, so that they can continue to provide the services. Because they had support networks that were going on, and what they were doing was actually housing people with families who are able to look after them as well, to provide them in their own communities … And I think if the councils were actually to look at the NGOs, because there are so many, and use them, use them to their advantage, they will get a better outcome and we won’t see these people on the streets, and at least eating a decent meal.”—P3, HCP*

To address ever-changing guidelines and the segmented nature of available support, participants recommended developing a resource that provides a clear pathway on how to care for individuals, based on their specific circumstances. An easy-to-follow framework or flowchart that signposts to various resources based on different eligibility, collating sources of local support and information, was suggested.

*“Sometimes I think I really need to look at some sort of pathway for NRPF so that midwives know what to do. Because at the moment it’s kind of haphazard. It's just like, “Oh, you know, we'll try this, and we'll try that. We'll, maybe ask [name] or safeguarding for some advice,” but it's haphazard. Whereas I think it's helpful for staff to have like a clear pathway - “Oh, you know, I don't know what to do with this lady. Oh, let me look at the policy”.—P8, HCP*

*“A really easy flowchart because so often we are really, really busy, so a flowchart that shows us that you’ve got the issue, no recourse to public funds, and then you could easily have evidence of domestic violence and that’s easy so then that’s down that route, or somebody on a spouse visa or whatever, just to make it really clear and easy as to who is eligible for what really.”—P9, HCP*

#### 3.2.3 Theme 3: systemic and legislative barriers

The final theme describes participants’ perspectives on what improvements needed to address systemic challenges that affect women and families with NRPF. These include stigma and negative perception in the community; lack of transparency and adequate legislative support from government; and NHS charging practices for maternity care, which discourage migrants from attending care or seeking medical help when needed.

##### 3.2.3.1 Dehumanised individuals

Participants discussed the dehumanising nature of NRPF, due to individuals’ rights have been stripped away. Structural policies and the broader hostile environment agenda led to exclusion and precarity, which directly impacted our participants and created a sense of hopelessness. They noted that this dehumanisation is further exacerbated by negative perceptions and stigma surrounding migrants, refugees, and asylum seekers, and social risk factors faced by this group such as homelessness, HIV, etc.

*“It’s such a false economy going after families with no money. I hate it if I’m honest.”—S3, HCP*

*“Things [support services and programs] are happening around you, but you can’t even try to apply for them. Or, you know when you’re in a place of work and you are even not treated fairly, but because you don’t have that right, you cease caring, there’s a limit to what you can fight … All these people know that you have limited things on your visa, and they use it against you.”—P14, Private care*

*“But because of the stigma of it all, it was very difficult for her to leave here to go there [in reference to being deported to home country, having a HIV positive status], because of ostracization more than anything else, from her community, so she remained that way. I got Maternity Action involved, and they came up with Section Four, because they were trying to say that she can fly back [to home country].”—P3, HCP*

##### 3.2.3.2 Need for changes to legislation

Professionals recognised the urgent need for legislative change at the national level, and the provision of better policy, guidelines and resources for services on the ground, in order to prevent further dehumanization. They emphasised that, to ensure pregnant migrant women and children are appropriately supported, local services have had to absorb costs and find alternative courses of action, which may end up costing more to the government in the long term.

*“The government has somehow passed on this hidden cost of pre-settled status, no recourse to public fund families on the local authority to bear … So I think the overarching problematic point is that there is a huge financial need that is demanded of the local authorities, but actually there is no funding from central to local authorities to make this happen.”—P5, LA*

*“I think this NRPF condition, going back to the immigration, I mean, just if the government would lift the NRPF condition because many people, they are here legally, … and they paid a lot of money for their visas to be here. And they want to contribute, as we just … I think we're all in agreement that people want to work and do something and contribute. But unfortunately, we have this condition imposed that ends up being, in my opinion from what I see in the Council, that is so much more expensive, at the end of the day, in terms of how much we spend to support people.”—P7, LA*

Participants also discussed a lack of transparency and acknowledgement of the issues faced by women and families as well as services, leading to lack of clear guidance and referrals, as well as vulnerable individuals being missed by services….

*“I mean, personally, my feeling is that NHS England have managed to remain very apolitical in terms of if you look at the equity and equality guidance there is absolutely no mention of people with no recourse to public funds, etc.”—P9, HCP*

*“It’s got to start hasn’t it really from central government, in the whole way that they look at vulnerable people. And from my perspective, women who are fleeing domestic abuse, whether they’re pregnant and will then need the services of people, it’s just one faction of that isn’t it. So how we’re dealing with them from a central government perspective would then need to filter out into local government. Because at the moment there just appears to be lots of silos, with different people doing different things, different people taking different stances, and trying to navigate that.”—P15, Charity*

Finally, participants highlighted how, immigration law surrounding NRPF being incredibly complex and ambiguous, compounded by the lack of available legal representatives in the sector who are knowledgeable about this type of law, impeded their ability to signpost women to the legal help they may require.

*“I suppose hearing that, I’m very conscious that somebody in [name] position, even if you know a fair bit about immigration and asylum yourself, would probably still have trouble finding a legal rep for somebody, just because of the state the sector’s in at the moment. So, I don’t think that this is fixable with the sector being as it is. I know that the government are trying to increase the asylum rates a little bit for legal aid, whether that will do anything or not, I don’t know. So immigration law is mostly out of scope for about the past 15 years, it was charities and stuff that were picking up the pieces.”—P16, Solicitor*

##### 3.2.3.3 Suggestions for improvement

While legislative change is necessary, perhaps removal of the NRPF wholly, to limit dehumanization of pregnant migrant individuals, participants reognised that this would require a systemic paradigm shift that may take years to enact. In lieu, they recognized that professionals could be better trained to understand and handle the complexities of NRPF and treat users with more compassion and empathy. They identified a need for training on the impact of immigration, particularly within maternity services, which should be incorporated into standard professional training mandatorily. This would improve professionals’ capacity and equip them with the tools to handle often difficult care interactions better.

*“But what I think would be useful is that like, the impact of immigration, that there’s kind of consideration to the impact of immigration in training courses. So I don't know if it's relevant for midwives, for example, but working with higher education institutes to kind of have a couple of hours where like, NRPF, in this professional context is considered and how you might deal with it, in the same way that you would consider other areas of practice.”—P11, LA*

*“… maybe one thing that would be really helpful is to have the confidence to know that the advice that I’m giving is not going to have implications for that person in terms of the Home Office. Because at the moment I think I’m quite nervous to advise, or I just don’t want to put my foot in it for a family.”—P12, HCP*

## 4 Discussion

### 4.1 Main findings

This study explored professionals’ perspectives on supporting pregnant women with No Recourse to Public Funds (NRPF) status in the UK who may be subject to NHS charging policies in England, including their views on existing policies and programs, knowledge gaps, training needs, and ideas to facilitate improved care. We used a mixed-method design comprising focus group discussions and online survey. Our results highlight significant and layered challenges faced by professionals providing care and support to pregnant women with NRPF. Quantitative data show low-to-moderate knowledge and confidence levels amongst professionals, aligning with the themes identified in focus groups, improving the robustness and credibility of the results. On a 0–100 scale, mean knowledge scores of immigration rules and regulation were 52.2 (SD = 27.3), 50.8 (SD = 30.0) for the NRPF visa condition specifically, 45.3 (SD = 28.1) for perinatal support available to these women, and 43.4 (SD = 29.0) for NHS maternity charging. Mean confidence was (57.1, SD = 36.2) in providing support to women with NRPF and 42.5 (SD = 32.5) for answering questions related to NHS charging, highlighting variability in professional preparedness. Although 88% wanted to improve their understanding of NRPF and NHS charging, only 14% had received prior training. Qualitatively, three themes were produced: (1) ineffective cross-sector communication, (2) lack of knowledge and resources, and (3) systemic and legislative barriers—each of which depicts a key challenge that may influence timely and appropriate support for pregnant migrants and their children. These challenges have direct implications for women and families: inconsistent guidance, delayed access to services, and fragmented support may contribute to financial hardship, increased stress, and potential adverse maternal and child health outcomes. They underscore the structural inadequacies and human costs associated with the current lack of support for this marginalised group.

### 4.2 Interpretation

These findings can be understood through the lens of the social determinants of health and structural vulnerability frameworks, which emphasise how health outcomes are shaped by intersecting social, economic, and policy conditions rather than individual-level factors alone. In this context, NRPF status operates as a structural determinant that constrains access to resources for service users, while simultaneously shaping how professionals are able to deliver care within institutional systems. Our findings highlight that cross-sector communication remains fragmented and inefficient, despite the multidisciplinary nature of care required for pregnant women with NRPF. Survey findings indicate that lower confidence in providing support is associated with perceptions of poor communication, supporting focus group reports that communication gaps were perceived to exacerbate coordination challenges, which in turn may help explain delays in care and suboptimal support for women and families. For instance, the absence of coordinated communication channels contributes to duplicated work, inefficiencies, and inappropriate referrals. These results align with wider concerns about siloed working across health and social care, particularly when caring for people with complex immigration-related needs (18, 19). Communication failures are not simply operational issues but are indicative of systemic fragmentation across health, social care, and immigration governance structures. Participants suggested a named key worker or liaison role, embedded within or across services, to facilitate inter-agency coordination. They stressed that this person would need to be a staff member, ideally of moderate seniority, already embedded in the system and familiar with its different components, rather than a new role. This echoes successful models of care coordination for individuals in other vulnerable situations (e.g., maternity care continuity models, ‘navigator’ roles provided by charities, or caseworker-led social care pathways) and would likely improve efficiency, reduce staff burden, and enhance continuity for service users (20, 21). The question of whether multidisciplinary working is hindered by a lack of formal structures, insufficient resources, or both, is an important area for future research. Previous studies (22) indicate that community-based care environments tend to better support multidisciplinary collaboration—especially around social determinants of health (23)—compared to larger hospital settings, where professionals may have less familiarity with local resources and services.

Gaps in professional knowledge and a lack of accessible resources further undermine service provision. The survey results mirror focus group findings, with mean knowledge scores of 43–52/100 reflecting reported uncertainty and inconsistent guidance, which may leave women unaware of available support or at risk of delayed or inadequate care. Focus group participants reported variable understanding of the NRPF condition and associated entitlements, with service access largely dependent on local experience, geographic location, and individual initiative. This variability in knowledge can be understood as a form of ‘institutionalised uncertainty’, produced by complex and frequently changing policies, which shift the onus of responsibility for interpretation onto frontline professionals.

A “postcode lottery” in services reflects wider inequalities in access to care and support for migrants across the UK (24). The alignment of survey data and qualitative reports demonstrates wide variation in knowledge and confidence, reinforcing the theme of geographically dependent service provision. The absence of clear, up-to-date guidance, together with under-resourced statutory and voluntary sectors, appears to leave professionals reliant on informal solutions, including personal donations and *ad hoc* charitable support. Such dependence on goodwill is unsustainable and risks both staff burnout and inconsistent care (25). Such working structures also add to moral injury, causing distress amongst professionals who are unable to provide the care required, and may feel complicit in the system causing harm (26). Participants strongly recommended a national pathway or decision-making tool to guide professionals in assessing needs and signposting to appropriate support, particularly in non-specialist or low-incidence areas. Implementing such tools could ensure women receive timely and appropriate support, potentially reducing stress, avoiding delayed interventions, and improving outcomes for mothers and infants. The survey findings, showing only 14% had received prior training, reinforce the need for formal guidance and evidence-based resources.

At a structural level, systemic and legislative constraints were highlighted as the most intractable and damaging. Immigration policies that enforce the NRPF condition were perceived by participants as undermining the wellbeing and rights of women and children, and as placing untenable burdens on local services without adequate funding or national oversight. Consistent with existing literature (27, 28), participants described a dehumanising environment in which women fear accessing care due to potential immigration consequences, stigma, NHS charging practices, and social care involvement. This fear was described to influence care-seeking behaviour with participants suggesting women may choose to delay or avoid care, increasing risks of maternal complications, adverse birth outcomes, and long-term impacts on child development (29, 30). Other work have found that the legislative exclusion of people with NRPF from safety nets and universal entitlements was also felt to create perverse economic consequences: short-term exclusion from support was believed to lead to greater expenditure downstream through emergency accommodation, safeguarding interventions, and crisis healthcare (31). Our findings reflect what has been described as a ‘hostile environment’ policy context in the UK, in which immigration enforcement is embedded within welfare and healthcare systems. From a structural perspective, this produces forms of systemic exclusion and a manifestation of structural violence, whereby policies indirectly harm health by restricting access to essential services.

A further concern was the lack of immigration-competent legal advice, which severely limits women’s ability to regularise their status or challenge unjust decisions. As others have noted (32, 33), the erosion of legal aid and the complexity of immigration law create a significant justice gap for people with NRPF. Participants noted that even where services sought to advocate on behalf of women, they lacked confidence in navigating the immigration system safely and legally. Survey data support this, as low confidence scores indicate professionals feel unprepared to advise women on NRPF or NHS charging, leaving women and families vulnerable to missed care, financial penalties, or stress. Mandatory training on the impact of immigration status in maternity and related professional education could help address this but cannot substitute for broader structural reform.

### 4.3 Strengths and limitations

This study has several strengths. It is the first study to systematically explore health and social care professionals’ perspectives on supporting pregnant women with NRPF status and NHS maternity charging. It draws on the perspectives of a diverse group of frontline professionals across sectors and regions, including midwives, social workers, charity workers, and public health practitioners. Their insights provide a grounded understanding of the practical and emotional toll of current policies and highlight evidence-based recommendations for improvement.

However, this study also has its limitations: The participant sample showed limited diversity in key demographics: most survey respondents were White (75%) and employed within the NHS (86%), with a majority having over 11 years of experience. Focus group participants were concentrated in London and a few other regions, and ethnic and professional diversity was limited. These factors may have influenced the perspectives captured, meaning the findings may not fully represent the experiences of all professionals across the UK, or globally. Future research should seek to gain a larger and more representative sample of the HCP population to examine how knowledge and attitudes differ between practitioners and how this may influence patient care. Furthermore, self-reported measures could have introduced biases due to poor recall and introspection.

Additionally, the study reflects professional perspectives only and does not include the voices of women with NRPF themselves. Further research is needed to centre lived experience and to evaluate the effectiveness of proposed interventions, such as liaison roles, professional training, decision-support tools and policy changes, in improving outcomes. Finally, those who actively engage with such initiatives are likely already motivated and informed, which highlights potential underlying systemic issues. The current findings may therefore represent just the tip of the iceberg. The larger NoRePF programme will further explore these questions through qualitative research focused on the lived experiences of migrant women with NRPF status and NHS charging, to inform potential future interventions and strategies to reduce maternal and child health inequalities. We also aim to investigate feasibility and effectiveness of implementing suggested training and guidance across sites in England (34).

## 5 Conclusion

This study highlights the need for coordinated national policy, investment in frontline services, and improved guidance for professionals supporting people with NRPF status in England. Without these measures, women and their families may be at risk of delayed care, financial strain, and increased stress, which can affect maternal and child health outcomes. Maternity services, in partnership with health, social care, and voluntary sector stakeholders, can play a key role in identifying needs early and advocating for safe, dignified care for all women and families.

This research shows sustainable improvements require multi-disciplinary collaboration, evidence-based training, and clear guidance, supporting professionals to provide timely and consistent care. Greater alignment between immigration policy, human rights, and public health objectives may help ensure access to essential services. Coordinated, deliberate action is essential to strengthen workforce preparedness, enhance service delivery, and mitigate risks for women and families affected by NRPF.

## Data Availability

The raw data supporting the conclusions of this article will be made available by the authors, without undue reservation.
