# Supplementary material for: Navigating No Recourse to Public Funds and NHS maternity charging: health and social care professionals’ experiences of supporting pregnant women in the UK
Source: Front Public Health. 2026 May 22;14:1772211. doi: 10.3389/fpubh.2026.1772211 (PMC13236894; doi:10.3389/fpubh.2026.1772211)
Supplement: Supplementary file 2 [file Supplementary_file_2.pdf]

NoRePF LA Mapping Focus Groups: Indicative Topic Guide

This is an indicative topic guide, and the final questions will be developed with the project Lived Experience Advisory Group.

Thank you for taking the time to be part of this focus group discussion in relation to the No Recourse to Public Funds (NoRePF) project. You have been invited to share your experiences today because you work with and support families with NRPF in your professional role.

Let me tell you a short summary of what we are doing. The NoRePF project, led by Dr Hannah Rayment-Jones at King's College London, aims to improve health and social outcomes for families and pregnant women with NRPF and develop multidisciplinary guidance for professionals who work with these individuals.

To do so, we first need to understand the existing knowledge base and need for further training and support. So today, I will ask you all a few questions about your work with NRPF families, what programs are available in your area of work, what you think are your knowledge gaps, and how you would like to be better supported or trained.

While I have interview questions to guide the discussion, since this a focus group, we would like to have an open and collaborative discussion.

The interview will be structured in 3 parts: (1) Current experience, (2) Existing programs and policies (3) Future training and support needs

We are interested in the full range of your experiences- there are no right or wrong answers, and you will not be judged based on what you say. This is in no way to catch you out or assess your performance in any way. Likewise, this will be completely anonymous, and you will in no way be reprimanded for anything you say here today.

It will last about an hour.

We can provide you with the transcript, should you wish.

Should you feel uncomfortable at any time and wish to leave or take a break, please tell me.

Do you have any questions before we begin? If not, I shall now start to record.

Just for the sake of recording, this is focus group number XX.

Section 1: Current experience

1. **Can you tell me about your day-to-day role and how it relates to supporting families and pregnant women with NRPF?**

   Probes:

   - Do you work closely with maternity services/ local authority/ social care (as relevant) in your local NHS Trusts?

Section 2: Existing programs and policies

1. **Are you aware of any programs and policies are available in your area to pregnant women and families with NRPF?**

   Probes:

- What programs are available in your area that you facilitate/ refer to?
- What policies are in place?
- Do you signpost people to childcare support? Any other allowances for childcare?
- Where do you refer people for more information?

2. **Do you know about the Healthy Start program and what is your experience in referring families to this?**
    - Do you help them complete the application?

Section 3: Future training and support needs

1. **Are you *currently* provided any training on working with NRPF families? If not, what kind of training would you like to be provided?**
    - Working with each other, across services/ colleagues in NRPF support?
2. **What else will help support you in your role?**

For women with NRPF, they are often charged to access routine maternity care, unclear and variable between Trusts.

3. **Do you know where to go/ send women for more information?**
4. **Should future guidance include information/recommendations about NHS Charging practices?**

Section 4: Final reflections

1. **Can you tell me about the overall barriers and facilitators for supporting pregnant women and families with NRPF?**
2. **In the multidisciplinary guidance this project aims to develop, what's one thing you would like to see included?**
3. **How would you like this guidance to be implemented?**
    - Online training/document pack/ face-to-face annual training update/CPD accreditation
4. **Any other reflections that you think would be important for us to hear?**

Thank you all very much for your time. We will send around vouchers as a small token of thanks for your time today. Bye!
